# Weight and weight changes throughout life and postmenopausal breast cancer risk: a case-control study in France

**DOI:** 10.1186/s12885-016-2793-0

**Published:** 2016-09-29

**Authors:** Emilie Cordina-Duverger, Thérèse Truong, Antoinette Anger, Marie Sanchez, Patrick Arveux, Pierre Kerbrat, Pascal Guénel

**Affiliations:** 1 Cancer & Environment Group, Center for Research in Epidemiology and Population Health (CESP), INSERM, University Paris-Sud, University Paris-Saclay, Villejuif, France; 2 Centre Georges-François Leclerc, Côte d’Or Breast Cancer Registry, Dijon, France; 3 Centre Eugène Marquis, Rennes, France

**Keywords:** Breast Cancer, Body Mass Index, Weight Gain, Case-control study

## Abstract

**Background:**

Overweight and weight gain throughout adult life have been associated with increased risk of breast cancer after the menopause. However the role of body weight at a young age and of the timing of weight gain over the lifetime in postmenopausal breast cancer is not well documented.

**Methods:**

We conducted a population-based case-control study on breast cancer in France that included 739 cases and 815 population controls in postmenopausal women. Height, weight at age 20, 40 and 50 as well as weight one year before diagnosis were obtained during in-person interviews.

**Results:**

No association between body mass index at the age of 20 years and breast cancer after the menopause was detected. However, we found that postmenopausal breast cancer was associated with weight gain between ages 40 and 50 years (OR per 5 kg/m2 increase in BMI: 1.45 [95%ci 1.06−1.98]). The increased risk of breast cancer associated with weight gain was more consistent in leaner women at age 20, in older postmenopausal women (>65 years), and in women who did not use menopausal hormone therapy.

**Conclusions:**

These findings point to the importance of controlling for weight gain in middle aged-women. The role of low body weight in young adulthood in breast cancer risk after the menopause should be further scrutinized.

**Electronic supplementary material:**

The online version of this article (doi:10.1186/s12885-016-2793-0) contains supplementary material, which is available to authorized users.

## Background

With over 1.5 million new cases each year across the world, breast cancer is the leading cause of cancer among women. Despite a recent decrease attributed to the reduction of menopausal hormone therapy, incidence rates of postmenopausal breast cancer in wealthy countries has grown steadily over the last decades. Rising incidence rates are also observed in emerging countries as high calorie intake and sedentary life become more common, pointing to the role of overweight and lack of physical activity as major modifiable causes of breast cancer among postmenopausal women.

The relationship between adiposity and breast cancer is complex and varies during lifetime. Before menopause, adiposity reduces the risk of breast cancer. This inverse association has been attributed to a decreased number of ovulations in overweight women and alteration of circulating hormone levels, which play a key role in breast cancer etiology, but other mechanisms may also account for the protective effect of high BMI before the menopause [1]. After the menopause, a high BMI increases the risk of breast cancer breast cancer, and this association is explained by estradiol production in the adipose tissue [2–4].

Investigating weight changes during lifetime, particularly in the period around the menopause, when the effect of BMI in breast cancer changes from a protective to a deleterious effect, is thus important to improve our understanding of the relationship between adiposity and breast cancer. However, most studies on breast cancer among postmenopausal women have only measured weight gain over long periods, usually from early adulthood to the time of cancer diagnosis, making difficult to evaluate how weight changes in specific periods of life may affect breast cancer risk [5–8].

Elevated BMI during childhood or adolescence has also been associated with a decreased risk of postmenopausal breast cancer in some studies [9–11]. It has been postulated that leanness in early adulthood may increase the risk of postmenopausal breast cancer due to incomplete differentiation of mammary gland cells related to insufficient mammary fat pad or progesterone deficiency [6, 12]. This association, however, should be scrutinized.

In order to clarify the relationship between postmenopausal breast cancer risk and lifetime weight history, we used the data of a large population-based case-control study in France, focusing on the timing of weight changes over the lifetime and on the role of low weight (BMI <18.5 kg/m2) at a young age.

## Methods

The CECILE study is a population-based case-control study in Côte d'Or and Ille-et-Vilaine, two French administrative areas (départements) located in Eastern and Western part of France, respectively.

### Recruitment of cases and controls

The case group included incident cases of in situ or invasive breast cancer diagnosed between April 2005 and March 2007 in women aged 25–75 years who resided in the study areas. Patients were recruited in the main cancer hospital in each area (Centre Eugène Marquis in Rennes and Centre Georges-François Leclerc in Dijon), as well as from smaller public and private hospitals that also recruited breast cancer patients. Among the 1553 eligible cases identified during the study period, 163 refused to participate, 151 could not be contacted, and 7 died before the interview, leaving 1232 cases included in the study sample (participation 79.3 %).

Controls were women without a previous history of breast cancer recruited in the general population of each study area. They were selected by phone and were frequency-matched to the cases by 10-year age group. To avoid selection bias that could arise from differential participation rates across categories of socioeconomic status (SES), we obtained a control group with a distribution by SES category similar to the general population, by using predefined numbers of controls by SES calculated from the census data. To recruit the controls, phone numbers of private homes were selected at random from the telephone directory completed beforehand with unlisted numbers. Phone numbers were dialed up to 15 times at different times of the day and different days of the week until contact could be established with the residents. When a woman was living in the residence reached by phone, she was invited to participate to the study, as long as the predefined number of controls in her age and SES stratum was not complete. When this number was exceeded, the woman was excluded. To obtain the desired number of controls within the limits of age and SES categories, approximately 30,000 phone numbers were dialed for identifying 1,731 eligible controls. Among 1731 controls identified by telephone fulfilling eligibility criteria, 260 declined participation and 154 could not be re-contacted for an in-person interview, leaving 1317 women available for the study (participation 76.1 %).

The study was approved by the French Ethic Committee (CCPPRB Kremlin-Bicêtre, Jan 2005), the National Data Protection Agency (Dec 2004) and the Advisory Committee on the Treatment of Health Research Information (Apr 2004). All participants signed informed consent.

### Selection of study subjects

Only postmenopausal women were included in the analysis. Women were considered postmenopausal if they had not menstruated for twelve or more months (natural menopause, *n* = 936), if they had had bilateral oophorectomy (artificial menopause, *n* = 93), or if they had used MHT (Menopausal Hormone Therapy) before natural cessation of menstruation (*n* = 352). Women with unknown menopausal status (*n* = 199) (hysterectomy before cessation of menstruations or unknown date of last menstruation), were considered postmenopausal if they were aged 50 or more years, the median age at menopause in women with natural menopause (*n* = 174). Women with unknown menopausal status below 50 years old were excluded from the analysis (*n* = 25). One woman reporting aberrant low weight was excluded from the analysis. In total, the analysis included 1554 postmenopausal women (739 cases and 815 controls).

### Data collection

Data pertaining to study subjects were obtained from a structured questionnaire during in-person interviews conducted by trained interviewers. We defined a reference date for each study subject, which was the date of diagnosis for the cases and the date of selection for the controls. The age at reference date will be referred to below as current age. Only events that occurred before that date were considered in the analyses. We elicited information on socio-demographic characteristics, history of previous diseases, family history of cancer, history of menstruations, use of oral contraceptives, infertility, reproductive history, residential and occupational history, lifetime consumption of alcohol and tobacco, recreational activities, and dietary habits.

Women were invited to report their height at the age of 20 years, and to report their usual weight one year before reference date (hereinafter referred to as current weight) to avoid reporting weight loss that might be due to cancer development. We also elicited information on weight at the ages of 20, 40 and 50 years.

Information on estrogen (ER) and progesterone receptor (PR) status was obtained from the pathology report. Tumors containing more than 10 % positive cells for hormonal receptors were classified as receptor-positive.

### Statistical analysis

Odds ratios (ORs) of breast cancer were calculated for BMI at age 20, BMI at age 50 and BMI at the current age, and for BMI changes from age 20 to current age, from age 20 to 40, from age 40 to 50, and from age 50 to current age. BMIs were calculated as weight in kilograms divided by height in meters squared (kg/m^2^). BMI at different ages were categorized according to the WHO classification (<18.5; 18.5–25; 25–30; ≥30 kg/m^2^), except for BMI at age 20 where we used a single category of BMI ≥ 25 kg/m^2^ due to small number of young obese women ≥30 kg/m^2^. BMI changes during different periods of life were categorized in 3 groups. To enable comparisons between BMI gain during different periods of life, and despite uneven distributions, we sought to use the same cut points for defining BMI gain categories in different periods: BMI gain <1 kg/m^2^; BMI gain ≥ 1 < 3 kg/m^2^; and BMI gain ≥ 3 kg/m^2^. For BMI changes from age 20 to current age, the highest category of BMI gain was subdivided in two classes (3–6 kg/m^2^ and ≥ 6 kg/m^2^). We also fitted models using tertiles or quartiles of BMI gain distribution among controls specific to each exposure period, but the findings were very similar and are not shown. To test dose-response trends, we fitted models where BMI at different ages and BMI changes were introduced as continuous variables, assuming a linear relationship between the variable and breast cancer risk, and reported odds ratios for each increment of 5 kg/m^2^ of BMI or BMI change.

Further analyses were conducted to examine whether low BMI at age 20 (<18.5; ≥ 18.5 kg/m^2^), age at reference date (<65, ≥ 65 years), and use of menopausal hormone therapy (MHT) (current vs past or never) modified the association of breast cancer with BMI at different ages, and BMI changes. In these analyses, we present only the odds ratios associated with continuous variables for BMI and BMI changes. *p*-values for interaction between BMI or BMI changes and the stratification variables (i.e. BMI at age 20 years, age at reference date and MHT use) were calculated by comparing models with and without an interaction term using the likelihood ratio test.

Odds ratios and 95 % confidence intervals were calculated using unconditional logistic regression models adjusting for the matching variables, i.e. age (5-year age group) and study area, and for breast cancer risk factors in Table 1: age at menarche (≤11, 12, 13, 14, ≥ 15 years), parity (0, 1, 2, 3, ≥ 4 children), age at first full-term pregnancy (<22, 22–24, 25–27, ≥ 28 years), duration of breast-feeding (0, <26, 26–52, >52 weeks), oral contraceptive use (ever, never), family history of breast cancer in first degree relatives (yes, no), MHT use (current, never or past use), recreational physical activity (ever/never), tobacco smoking (never, < 10, ≥ 10 packs-years), and alcohol consumption (≤3, 4–7, 8–14, > 14 glasses per week).

**Table 1:** Distribution of cases and controls by age, study area and selected risk factors of breast cancer

	Cases (*n* = 739)		Controls (*n* = 815)		OR^a^	95 % CI
	N	%	N	%		
Study area (département)						
Côte d'Or	235	31.8	284	34.8		
Ille-et-Vilaine	504	68.2	531	65.2		
Age at reference date (years)						
35-39	0	0.0	1	0.1		
40-44	1	0.1	1	0.1		
45-49	18	2.4	33	4.1		
50-54	103	13.9	109	13.4		
55-59	189	25.6	192	23.6		
60-64	158	21.4	164	20.1		
65-69	147	19.9	192	23.6		
70-74	123	16.6	123	15.1		
Family history of breast cancer in first degree relatives						
No	604	81.7	715	87.7	1	ref
Yes	135	18.3	100	12.3	1.62	[1.22-2.16]
Age at menarche (years)						
≤ 11	131	18	122	15.2	1	ref
12	179	24.7	172	21.4	0.98	[0.71-1.35]
13	155	21.3	174	21.6	0.82	[0.59-1.15]
14	143	19.7	165	20.5	0.81	[0.58-1.13]
≥ 15	118	16.3	172	21.4	0.63	[0.45-0.89]
Parity						
Nulliparous	79	10.7	50	6.1	1	ref
1 FTP^b^	109	14.7	113	13.9	0.61	[0.39-0.95]
2 FTP	279	37.8	270	33.1	0.65	[0.44-0.96]
3 FTP	183	24.8	245	30.1	0.46	[0.31-0.69]
≥ 4 FTP	89	12	137	16.8	0.41	[0.26-0.64]
Age at first FTP among parous women (years)						
< 22	185	28	252	32.9	1	ref
22-24	196	29.7	252	32.9	1.07	[0.82-1.40]
25-27	141	21.4	167	21.8	1.16	[0.86-1.56]
> 27	138	20.9	94	12.3	2.02	[1.46-2.81]
Breastfeeding among parous women (weeks)						
never	349	53.5	399	52.2	1	ref
< 26	224	34.4	261	34.2	1.03	[0.82-1.31]
26-52	54	8.3	67	8.8	0.97	[0.66-1.43]
> 52	25	3.8	37	4.8	0.82	[0.48-1.40]
Age at menopause (years)^c^						
< 48	105	22.7	115	22.9	1	ref
48-50	136	29.4	157	31.2	0.85	[0.59-1.23]
51-53	133	28.8	126	25	1.03	[0.71-1.50]
≥ 54	88	19	105	20.9	0.82	[0.54-1.23]
Current MHT use						
No	589	79.7	690	84.7	1	ref
Yes	150	20.3	125	15.3	1.44	[1.10-1.88]
Height (cm)						
< 158	187	25.4	230	28.2	1	ref
158-161	188	25.5	198	24.3	1.25	[0.94-1.67]
162-165	172	23.3	223	27.4	1.03	[0.77-1.38]
≥ 166	190	25.8	164	20.1	1.63	[1.21-2.20]
Alcohol consumption (glasses per week)						
≤ 3	542	73.3	581	71.3	1	ref
4-7	108	14.6	122	15.0	0.92	[0.69-1.22]
8-14	53	7.2	71	8.7	0.77	[0.52-1.12]
> 14	36	4.9	41	5.0	0.91	[0.57-1.45]
Tobacco (pack-years)						
Never	500	68.3	563	70.2	1	ref
< 10	119	16.3	134	16.7	1.03	[0.77-1.36]
≥ 10	113	15.4	105	13.1	1.28	[0.95-1.74]
Physical activity						
No	269	36.8	264	32.5	1	ref
Yes	462	63.2	548	67.5	0.83	[0.67-1.02]

^a^Odds ratios adjusted for age at reference date and study area

^b^FTP: Full-Term Pregnancy

^c^Age at menopause unknown in 227 cases and 312 controls

All analyses were also conducted using different categorization for BMI and BMI gain during lifetime, or using weight and weight changes (in kg) instead of BMI. These analyses produced similar findings and are not reported here. We also conducted analyses stratifying the case group according to hormone receptor status of the tumor (ER-positive/ER-negative, PR-positive/PR-negative) using polytomous logistic regression models, but no particular hint emerged from this analysis (not shown).

All analyses were conducted using SAS computer software (version 9.3, Cary, North Carolina).

## Results

Selected characteristics of cases and controls are shown in Table 1. As expected from frequency-matching, the distributions by age and study area were similar for cases and controls. Breast cancer was associated with family history of breast cancer in first-degree relatives, early age at menarche, low parity, late age at first full-term pregnancy, current use of MHT, height and physical activity. Cases and controls did not differ in our data with respect to duration of breastfeeding, age at menopause, alcohol or tobacco consumption.

Table 2 shows mean BMI at age 20, mean BMI at age 50 and mean BMI changes between ages 20 and 40, 40 − 50 and 50 to current age after stratification of the control group by category of current BMI. There was a clear trend of higher BMI at ages 20 and 50 and of higher BMI gain as current BMI becomes higher. Table 2 also shows the Pearson’s correlation coefficients with current BMI. Correlations were moderate for BMI at age 20 and BMI change between ages 40 and 50 (Pearson’s r = 0.32), intermediate for BMI changes between ages 20 and 40 and BMI changes between age 50 and current age (Pearson’s r = 0.54 and 0.55, respectively), and strong for BMI at age 50 (Pearson’s r = 0.79).

**Table 2:** Mean values of BMI at different ages and of BMI changes by categories of current BMI among controls

	Current BMI (kg/m^2^)				
	<25	25-30	≥30	*p* ^a^	Pearson r^b^
	(*n* = 428)	(*n* = 245)	(*n* = 141)		
Mean current BMI (kg/m2) (sd)	22.0 (1.9)	27.2 (1.4)	34.2 (4.2)	<10^-4^	1.00
Mean BMI at age 50 (kg/m2) (sd)	21.4 (2.3)	24.7 (2.6)	29.8 (4.6)	<10^-4^	0.79
Mean BMI at age 20 (kg/m2) (sd)	20.1 (2.3)	20.9 (2.6)	22.5 (3.2)	<10^-4^	0.32
Mean BMI change from age 20 to current age (kg/m2) (sd)	1.9 (2.8)	6.3 (2.9)	11.8 (5.1)	<10^-4^	0.86
Mean BMI change from age 20 to 40 (kg/m2) (sd)	0.6 (2.4)	2.3 (2.3)	4.6 (4.7)	<10^-4^	0.54
Mean BMI change from age 40 to 50 (kg/m2) (sd)	0.6 (1.7)	1.5 (2.2)	2.9 (4.4)	<10^-4^	0.32
Mean BMI change from age 50 to current age (kg/m2) (sd)	0.6 (2.0)	2.5 (2.5)	4.5 (4.7)	<10^-4^	0.55

^a^ *P*-value of ANOVA

^b^Pearson correlation coefficient with current BMI

After adjustment for potential confounders listed in Table 1, BMI at age 20, BMI at age 50 and current BMI were not found to be associated with postmenopausal breast cancer (Table 3). However, BMI gain between 40 and 50 years of age was associated with increased risk of breast cancer (OR per 5 kg/m^2^ BMI gain between ages 40 and 50: 1.32; 95 % ci 1.05–1.65). Further adjustment for current BMI did not modify this finding. No association was observed with BMI gain before 40 and after 50 years of age. Models were also fitted using weight changes in kg instead of BMI in kg/m2. The results are shown in Additional file 1: Table S1 and yielded similar conclusions.

**Table 3:** Odds ratios of postmenopausal breast cancer for BMI at age 20, age 50 and current age, and for BMI gain from age 20 to current age, age 20 to 40, age 40 to 50, and age 50 to current age par category and per increment of 5 kg/m^2^ of BMI or BMI gain

	Cases		Controls		OR^a^	95 % CI	p trend^b^
	(*n* = 739)		(*n* = 815)				
	N	%	N	%			
BMI at age 20 (kg/m^2^)							
< 18.5	146	20.3	150	19.0	1.10	[0.84-1.44]	
≥ 18.5 < 25	527	73.2	587	74.2	1	ref	
≥ 25	47	6.5	54	6.8	0.92	[0.60-1.41]	
*Per 5 kg/m* ^*2*^					*1.03*	*[0.85-1.25]*	*0.76*
BMI at age 50 (kg/m^2^)							
< 18.5	34	4.9	29	3.8	1.14	[0.67-1.96]	
≥ 18.5 < 25	461	65.8	510	66.6	1	ref	
≥ 25 < 30	147	21.0	162	21.1	1.07	[0.81-1.40]	
≥ 30	59	8.4	65	8.5	1.05	[0.70-1.56]	
*Per 5 kg/m* ^*2*^					*1.07*	*[0.94-1.22]*	*0.30*
Current BMI (kg/m^2^)							
< 18.5	15	2.0	21	2.6	0.73	[0.36-1.50]	
≥ 18.5 < 25	374	50.7	407	50.0	1	ref	
≥ 25 < 30	223	30.3	245	30.1	1.03	[0.80-1.31]	
≥ 30	125	17.0	141	17.3	1.04	[0.77-1.41]	
*Per 5 kg/m* ^*2*^					*1.03*	*[0.92-1.14]*	*0.62*
BMI change from age 20 to current age (kg/m^2^)							
< 1	139	19.3	161	20.4	1	ref	
≥ 1 < 3	140	19.4	136	17.2	1.18	[0.83-1.66]	
≥ 3 < 6	196	27.2	211	26.7	1.10	[0.81-1.51]	
≥ 6	245	34.0	283	35.8	1.06	[0.78-1.44]	
*Per 5 kg/m* ^*2*^					*1.02*	*[0.91-1.14]*	*0.76*
BMI change from age 20 to 40 (kg/m^2^)							
< 1	306	44.0	338	43.8	1	ref	
≥ 1 < 3	240	34.5	232	30.1	1.22	[0.95-1.57]	
≥ 3	150	21.6	202	26.2	0.88	[0.66-1.16]	
*Per 5 kg/m* ^*2*^					*0.88*	*[0.74-1.06]*	*0.18*
BMI change from age 40 to 50 (kg/m^2^)							
< 1	329	48.2	396	52.7	1	ref	
≥ 1 < 3	223	32.7	234	31.2	1.18	[0.92-1.51]	
≥ 3	131	19.2	121	16.1	1.45	[1.06-1.98]	
*Per 5 kg/m* ^*2*^					*1.32*	*[1.05-1.65]*	*0.02*
BMI change from age 50 to current age (kg/m^2^)							
< 1	310	44.2	321	41.9	1	ref	
≥ 1 < 3	212	30.2	240	31.3	0.95	[0.73-1.23]	
≥ 3	179	25.5	205	26.8	0.97	[0.73-1.29]	
*Per 5 kg/m* ^*2*^					*1.00*	*[0.83-1.21]*	*0.97*

^a^Odds ratio adjusted for study area, age at reference date, age at menarche, parity, age at first full-term pregnancy, breastfeeding, family history of breast cancer, oral contraceptive use, current use of MHT, alcohol consumption, tobacco smoking, and physical activity

^b^p for trend calculated from the model using BMI or BMI change as continuous variables

Results of the stratification of BMI and BMI gain variables by BMI at age 20 (<18.5 kg/m^2^; ≥18.5 kg/m^2^), current age (<65 years; ≥65 years), and MHT use (current users; non-current users) are shown in Table 4. The stratification by BMI at age 20 showed that among leaner women at age 20 (BMI 20 < 18.5 kg/m^2^), the odds ratios for each increment of 5 kg/m^2^ of current BMI (OR 1.47; 95 % ci 1.05–2.07) and of BMI-gain between ages 40 and 50 (OR 2.06; 95 % ci 1.09–3.88) were markedly higher than the corresponding odds ratios in women with BMI at age 20 ≥ 18.5 kg/m^2^. However the *p*-values for interaction between BMI at age 20 and current BMI or BMI gain were not statistically significant (p interaction 0.14 and 0.40, respectively). The stratification by current age showed higher odds ratios for BMI at age 50, current BMI and BMI gain between 40 and 50 years in women ≥ 65 years than in women <65 years, with *p*-values for interaction 0.07, 0.08 and 0.08, respectively. Finally, the stratification on MHT use showed that the odds ratio for BMI gain between ages 40 and 50 was 1.37 (95 % ci 1.08–1.74) in MHT non-users whereas it was 1.02 (95 % ci 0.47–2.20) in current users (*p*-value for interaction 0.34).

**Table 4:** Odds ratios per 5 kg/m^2^ increase of BMI at age 20, BMI at age 50, current BMI and BMI changes stratified by BMI at age 20, current age and current MHT use

	BMI at age 20 < 18.5 kg/m^2^			BMI at age 20 ≥ 18.5 kg/m^2^			*p* interaction
	(146 cases / 150 controls)			(574 cases / 641 controls)			
	OR^a^	95 % CI	*p* trend^b^	OR^a^	95 % CI	*p* trend^b^	
BMI at age 50	1.36	[0.88-2.01]	0.16	1.08	[0.93-1.25]	0.31	0.52
Current BMI	1.47	[1.05-2.07]	0.02	0.99	[0.88-1.12]	0.91	0.14
BMI gain, age 20-40	1.05	[0.60-1.85]	0.85	0.85	[0.70-1.03]	0.09	0.46
BMI gain, age 40-50	2.06	[1.09-3.88]	0.02	1.29	[1.00-1.65]	0.05	0.40
BMI gain, age 50 to current age	1.62	[0.96-2.74]	0.06	0.94	[0.76-1.15]	0.53	0.28
	Age < 65 y			Age ≥ 65 y			*p* interaction
	(469 cases / 500 controls)			(270 cases / 315 controls)			
	OR^a^	95 % CI	p trend^﻿b﻿^	OR^a^	95 % CI	p trend^﻿b﻿^	
BMI at age 20	1.04	[0.82-1.33]	0.74	1.07	[0.77-1.47]	0.69	0.76
BMI at age 50	1.03	[0.88-1.21]	0.68	1.27	[0.98-1.63]	0.06	0.07
Current BMI	0.97	[0.84-1.11]	0.66	1.18	[0.98-1.42]	0.08	0.08
BMI gain, age 20-40	0.83	[0.67-1.04]	0.10	1.06	[0.74-1.50]	0.75	0.20
BMI gain, age 40-50	1.24	[0.95-1.61]	0.11	1.88	[1.14-3.07]	0.01	0.08
BMI gain, age 50 to current age	0.97	[0.74-1.27]	0.82	1.04	[0.80-1.36]	0.76	0.87
	Current MHT users			Non MHT users			*p* interaction
	(150 cases / 125 controls)			(589 cases / 690 controls)			
	OR^a^	95 % CI	p trend^ b^	OR^a^	95 % CI	p trend^﻿b﻿^	
BMI at age 20	0.97	[0.54-1.74]	0.91	1.03	[0.84-1.26]	0.81	0.81
BMI at age 50	1.05	[0.69-1.60]	0.81	1.07	[0.93-1.24]	0.31	0.75
Current BMI	1.08	[0.77-1.50]	0.65	1.03	[0.92-1.16]	0.63	0.90
BMI gain, age 20-40	1.27	[0.72-2.24]	0.40	0.86	[0.70-1.04]	0.13	0.73
BMI gain, age 40-50	1.02	[0.47-2.20]	0.97	1.37	[1.08-1.74]	0.01	0.34
BMI gain, age 50 to current age	1.06	[0.59-1.88]	0.85	1.00	[0.82-1.22]	0.99	0.69

^a^ORs adjusted for study area, age, age at menarche, parity, age at first full-term pregnancy, breastfeeding, family history of breast cancer, OC use, current use of MHT (where appropriate), alcohol consumption, tobacco smoking, and physical activity

^b^p for trend calcul﻿ated from the model using BMI or BMI change as continuous variables

Analyses were also conducted by tumor subtypes defined according to hormonal receptor status (ER-positive, ER-negative, PR-positive, PR-negative). No difference between tumor subtypes was observed (data not shown).

## Discussion

We found that weight gain in the age range period 40–50 years, but not in earlier periods of life, was associated with increased risk of postmenopausal breast cancer. Conversely, our data do not confirm that postmenopausal breast cancer risk is increased in women with low BMI at a young age. However, the association between weight gain between 40 and 50 and postmenopausal breast cancer risk, was more consistent among leaner women at the age of 20, among older women (≥65 years at diagnosis) and among non-MHT users. These findings point to the importance of examining weight history over the lifetime to clarify the relationships between adiposity and breast cancer risk after the menopause.

### Weight gain

BMI gain in adulthood has been linked to the risk of postmenopausal breast cancer in previous investigations [5, 8, 10, 13]. Although the timing of weight gain during life may be an important determinant of breast cancer risk, epidemiological evidence is sparse since most studies have assessed weight gain over long periods from early adulthood to the time of cancer diagnosis regardless of specific time periods [5, 8, 9, 14]. Our results suggest that weight gain during the age range period 40–50 years, i.e. in late reproductive period, may be particularly harmful. These findings are consistent with studies that reported increased risk of breast cancer among women who gained weight in middle adulthood [15, 16]. If this is confirmed, it would point to the importance of controlling weight gain in that period of life.

We also observed that the association of breast cancer with current BMI and weight or BMI gain in the age range period 40–50 years was stronger in women above 65 years of age than in younger postmenopausal women, suggesting a relatively long induction period between weight gain and breast cancer occurrence. Alternatively, it is possible that the beneficial effect of adiposity during pre-menopause may compensate the adverse effect of overweight in early post-menopause.

### Weight in early adulthood

The hypothesis that weight during adolescence or young adulthood may influence breast cancer risk after the menopause is supported by several epidemiological studies reporting an inverse association between weight at a young age and postmenopausal breast cancer [9–11, 17, 18]. In addition, it was demonstrated that pre-pubertal girls with low weight have higher mammographic density when they become adults [19], and mammographic density is one of the strongest risk factors for breast cancer [20]. It was also postulated that low level of adiposity in the mammary gland may alter breast tissue maturation, making breast tissue more susceptible to carcinogenic stimuli among leaner women [6, 21]. Our data did not confirm the hypothesis of a direct link between low weight at a young age and breast cancer risk after menopause, as no association between BMI at age 20 and postmenopausal breast cancer was detected. However, there was weak indication that weight gain between age 40 and 50 might lead to higher postmenopausal breast cancer risk in women who were leaner at a young age (BMI < 18.5 kg/m^2^). This finding should be interpreted with care as no statistically significant interaction between BMI at age 20 and BMI gain was seen. Nevertheless, it is consistent with a report from the large US Nurse’s Health Study showing that the association of postmenopausal breast cancer risk with weight gain of 25 kg or more since the age 18 years was stronger in women with BMI below 21 kg/m^2^ at age 18 years than in heavier women (p for interaction 0.05) [13]. This result points to the importance of examining lifelong weight history in order to elucidate the complex relationships between adiposity and breast cancer risk.

### Current MHT use

The association of postmenopausal breast cancer risk with weight gain between ages 40 and 50 years was apparent only among non-current MHT users, although the interaction between BMI gain and MHT use was not significant. This is consistent with several studies that reported an association between adiposity and postmenopausal breast cancer only among women who did not use MHT [5, 6, 8, 10, 13, 18, 22–24]. To explain this frequent observation, it has been suggested that the increased levels of circulating estrogens in women treated with hormones are predominant and mask the effects of adiposity on breast carcinogenesis [25].

### Study strengths and limits

In our study, incident breast cancers were identified on a population basis in well-defined geographical areas, using inclusion criteria similar to a cancer registry, and using active real-time search in the main cancer hospitals in each area. Controls were carefully selected from the study base controlling for possible differential participation rates across SES categories. In addition, all potentially important confounders were taken into account in the analysis.

The main limitations of the study include the self-reported and recalled history of height and weight. Studies that examined the accuracy of self-reported height and weight compared to measured values consistently reported that height tended to be overestimated and weight to be underestimated by the women [26–28]. If this applies to our study, then BMI values should be underestimated. Errors due to recalled weight at younger ages are also likely to have occurred, particularly for longer recall [29]. However, we think that misclassification errors due to self-reported or recalled weight were most likely non-differential, and are not probable explanations for the observed associations. Indeed, the cases and the controls were interviewed in the same way using a standardized questionnaire, they were not aware of the specific objectives of the study, and the possible link of weight changes with breast cancer is not a widely known fact among women in France. Moreover the main findings of our study were in line with expectations. As in other studies, chance findings may have occurred especially as we performed a large number of tests. Conversely some associations may have remained undetected due to low statistical power in some analyses. In particular, statistical power was limited to detect interactions between variables. With these limitations in mind, however, we believe that these data are of valuable interest given the scarcity of studies examining the effect of BMI assessed over the lifetime on breast cancer risk, and provide further opportunities for research.

## Conclusion

Weight history throughout life appears to be a key determinant of breast cancer risk after the menopause, but interplay between age, weight, weight gain and breast cancer risk factors appears to be complex. Our findings point to the importance of controlling for weight gain in middle aged-women. The role of low body weight during early adulthood in postmenopausal breast cancer risk should be examined further.

## Additional file

Additional file 1: Table S1. Odds ratios of postmenopausal breast cancer for weight gain (in kg) from age 20 to current age, age 20–40, age 40–50, and age 50 to current age per increment of 10 kg of weight gain. (DOCX 24 kb)

## Abbreviations

BMI: Body mass index
ER: Estrogen receptor
MHT: Menopausal hormone therapy
OR: Odds ratios
PR: Progesterone receptor
SES: Socio-economic status
