# Supplementary material for: Weight and weight changes throughout life and postmenopausal breast cancer risk: a case-control study in France
Source: BMC Cancer. 2016 Sep 29;16:761. doi: 10.1186/s12885-016-2793-0 (PMC5041340; doi:10.1186/s12885-016-2793-0)
Supplement: Additional file 1: Table S1. — Odds ratios of postmenopausal breast cancer for weight gain (in kg) from age 20 to current age, age 20–40, age 40–50, and age 50 to current age per increment of 10 kg of weight gain. (DOCX 24 kb) [file 12885_2016_2793_MOESM1_ESM.docx]

**Table S1: Odds ratios of postmenopausal breast cancer for weight gain (in kg) from age 20 to current age, age 20 to 40, age 40 to 50, and age 50 to current age par category and per increment of 10 kg of weight gain**

|  |  | **Cases** | | **Controls** | | **OR^[[1]](#footnote-1)^** | **95% CI** | **p trend** |
| --- | --- | --- | --- | --- | --- | --- | --- | --- |
|  |  | **(n=739)** | | **(n=815)** | |  |  |  |
|  | | **N** | **%** | **N** | **%** |  |  |  |
|  | **Weight change, age 20 to current age (kg)** | | | | | |  |  |
|  | <2 | 128 | 17.8 | 153 | 19.3 | 1 | ref |  |
|  | ≥2 < 8 | 161 | 22.3 | 151 | 19.1 | 1.26 | [0.90-1.78] |  |
|  | ≥8 < 16 | 196 | 27.2 | 233 | 29.5 | 1.06 | [0.77-1.46] |  |
|  | ≥16 | 236 | 32.7 | 254 | 32.1 | 1.17 | [0.86-1.61] |  |
|  | *Per 10 kg* |  | |  | | *1.02* | *[0.94-1.12]* | *0.59* |
|  | **Weight change from age 20 to 40 (kg)** | | | | |  |  |  |
|  | <2 | 258 | 37.0 | 265 | 34.3 | 1 | ref |  |
|  | ≥2 < 8 | 282 | 40.5 | 299 | 38.7 | 1.03 | [0.80-1.32] |  |
|  | ≥8 | 157 | 22.5 | 208 | 26.9 | 0.83 | [0.62-1.11] |  |
|  | *Per 10 kg* |  | |  | | *0.91* | *[0.79-1.04]* | *0.91* |
|  | **Weight change from age 40 to 50 (kg)** | | | | |  |  |  |
|  | <2 | 262 | 38.3 | 315 | 41.9 | 1 | ref |  |
|  | ≥2 < 8 | 286 | 41.8 | 308 | 41.0 | 1.14 | [0.89-1.46] |  |
|  | ≥8 | 136 | 19.9 | 128 | 17.0 | 1.42 | [1.03-1.94] |  |
|  | *Per 10 kg* |  | |  | | *1.25* | *[1.05-1.49]* | *0.01* |
|  | **Weight change from age 50 to current age (kg)** | | | | | |  |  |
|  | <2 | 287 | 40.9 | 304 | 39.7 | 1 | ref |  |
|  | ≥2 < 8 | 259 | 36.9 | 277 | 36.2 | 1.02 | [0.80-1.32] |  |
|  | ≥8 | 156 | 22.2 | 185 | 24.2 | 0.96 | [0.71-1.28] |  |
|  | *Per 10 kg* |  | |  | | *1.02* | *[0.88-1.18]* | *0.79* |

1. Odds ratio adjusted for study area, age at reference date, age at menarche, parity, age at first full-term pregnancy, breastfeeding, family history of breast cancer, oral contraceptive use, current use of MHT, alcohol consumption, tobacco smoking, and physical activity. [↑](#footnote-ref-1)
